# Mechanical dyssynchrony and diastolic dysfunction are common in LVH: a pilot correlation study using Doppler echocardiography and CZT gated-SPECT MPI

**DOI:** 10.1038/s41598-018-22213-z

**Published:** 2018-03-08

**Authors:** Szu-Ying Tsai, Shan-Ying Wang, Yu-Chien Shiau, Yen-Wen Wu

**Affiliations:** 1 Department of Nuclear Medicine, Far Eastern Memorial Hospital, New Taipei City, Taiwan; 2 Department of Biomedical Imaging and Radiological Sciences, National Yang-Ming University, Taipei, Taiwan; 3 National Yang-Ming University School of Medicine, Taipei, Taiwan; 4 Department of Nuclear Medicine, National Taiwan University Hospital and National Taiwan University College of Medicine, Taipei, Taiwan

## Abstract

Hypertrophic cardiomyopathy (HCM) is an often under-diagnosed cause of left ventricular hypertrophy (LVH). It affects 1/500 of the population, is the most commonly inherited cardiovascular disorder, and can present in apical, concentric, or septal forms. Although most patients are asymptomatic, sudden cardiac death can be the initial presentation of HCM. By retrospectively enrolling patients suspected of having three different types of HCM in the absence of epicardial coronary stenosis, we aimed to examine systolic and diastolic dysfunction and perfusion abnormalities using both Doppler echocardiography and state-of-the-art gated single-photon emission computerized tomography (SPECT) myocardial perfusion imaging (MPI) with a cadmium-zinc-telluride camera and thallium-201. Both regional perfusion and gated SPECT parameters were collected in addition to diastolic parameters from Doppler echocardiography. The results showed that mild ischemia was common in patients suspected of having HCM, with a mean summed stress score of 4.7 ± 4.9 (score 0–4 in 17-segment model). The patients with HCM were associated with discernible left ventricular mechanical dyssynchrony, especially those with the apical form. In addition, diastolic dysfunction was prevalent and early to late ventricular filling velocity ratios were significantly different between groups. By combining gated-MPI and Doppler data, the trivial functional changes in HCM may be identified.

## Introduction

Left ventricular hypertrophy (LVH) is a well-known independent risk factor associated with a high risk of adverse outcomes^1,2^. The common causes include hypertrophic cardiomyopathy (HCM) and hypertension^3^, while obesity, aortic stenosis and chronic kidney disease can also contribute to left ventricular (LV) wall thickness^4–6^.

HCM is an often under-diagnosed disease^7^, with an estimated prevalence of approximately 1/500 of the general population. It is equally present in both men and women, and can affect any age group^8,9^. The clinical diagnosis of HCM is predicated upon an increased LV wall thickness, particularly within the interventricular septum, the magnitude of which cannot be explained by other cardiac or systemic diseases^10–12^. Various types of sarcomere gene mutations have been identified in more than half of the patients with HCM^13–15^. Although most patients are asymptomatic and remain disability-free with a normal or near-normal life expectancy^9,16^, symptoms including shortness of breath, chest pain, syncope and palpitations can occur. In some patients, sudden cardiac death (SCD) can be the initial presentation of HCM^9^.

For adult patients, the typical cutoff value for HCM is an LV wall thickness ≥15 mm. Patients with a wall thickness ≥13 mm may be considered diagnostic, especially when other relevant information such as family history, non-cardiac symptoms and signs, or electrocardiogram abnormalities are present^10,11^. Three groups of HCM phenotypes have been identified, including septal, concentric and apical forms^17^. The septal form accounts for more than half of the cases of the disease, with echocardiography features of a septal to lateral wall thickness ratio ≥1.3^18^. The apical form is relatively rare, and is more prevalent in Asians^18–20^.

Hypertensive cardiovascular disease (HCVD) is another common cause of LVH^21–23^, in which the LV wall thickness is usually normal or ≤13 mm, and usually results in concentric remodeling/hypertrophy in most hypertensive patients^21,24,25^. However, HCM can coexist with hypertension, leading to difficulty in differentiating it from hypertensive heart disease^21,23^. Compared to HCVD, patients with HCM are associated with a thicker right ventricular wall, greater ST-T changes, lower LV mass and fibrosis, and decreased tricuspid annular motion velocity early diastolic velocity (TAM-e′)^21,22^. The patients with comorbid HCM and hypertension tend to be older, and have higher risks of diabetes, coronary artery disease, and non-cardiac death^23^.

Myocardial ischemia devoid of epicardial coronary stenosis can be seen in patients with HCM^15^, which originates from microvascular dysfunction with severely blunted coronary flow reserve, as well as widespread myocyte remodeling and myocardial disarray^15,26^. Stress single-photon emission computed tomography (SPECT) myocardial perfusion imaging (MPI) is the most commonly used noninvasive approach to evaluate ischemia, and up to 25% of patients with HCM can be detected with either reversible or fixed defects^10,27^. Abnormal perfusion on stress MPI can predict long-term survival and identify patients with a poor prognosis^27^. Both systolic and diastolic dysfunction can be observed in patients with HCM, and LV relaxation and diastolic filling usually precede the systolic abnormalities^15^.

Electrocardiogram-gated SPECT (GSPECT) images can provide comprehensive information regarding perfusion and LV function^28,29^. Conventional scanners indirectly convert gamma rays to an electric signal, and consist of Anger cameras (comprised of thallium-activated sodium iodide (NaI [Tl]) crystals) and photomultiplier tubes (PMTs). A conventional SPECT scan usually requires 10 to 20 minutes for acquisition with an average spatial resolution of approximately 10–20 mm^30^. In contrast, a high-speed camera using cadmium zinc telluride (CZT) detectors directly converts gamma radiation to an electric signal with improved spatial resolution and count rates, resulting in a lower injected dose and reduced scanning time to approximately 5 minutes^31^. As a consequence of the use of CZT cameras for MPI, GSPECT has become a tool used in everyday practice.

The goals of this study were to evaluate dipyridamole-induced abnormalities in patients with patent coronary arteries and suspected of having HCM, and to analyze the patterns of perfusion and functional aberration by GSPECT CZT MPI and Doppler echocardiography.

## Results

Fifty patients with suspected HCM were included in this study, and their characteristics are shown in Table 1. The patients’ mean age was 59.7 years ±12.4 years, and 50.0% (n = 25) of the study population was male. More than half of the patients had the concentric form (52.0%, n = 26), 34.0% (n = 17) had the septal form, and 14.0% (n = 7) had the apical form. Due to the retrospective nature of the study, most patients did not have sufficient information regarding their family history. According to the medical records, seven patients (14.0%) experienced unexplained syncope. Only one patient had non-sustained ventricular tachycardia, and expired two days after cardiac angiography. The calculated 5-year risk of SCD based on existing information was 1.484 ± 1.031(%); most patients (n = 47, 94.0%) had a low risk (risk < 4%), and three patients (6.0%) had an intermediate risk (risk ≥ 4% but < 6%), indicating relatively mild disease in our included cases. Most patients (76.0%, n = 38) were comorbid with hypertension, and 56.0% (n = 28) were either current or ex-smokers. There were no statistically significant differences in age, gender, comorbid hypertension or 5-year risk of SCD between the three forms (Table 2).

**Table 1:** Characteristics of all enrolled patients (n = 50).

Clinical Variables	
Age (y)	59.7 ± 12.4
Male gender	25 (50.0)
Type of HCM	
Apical	7 (14.0)
Concentric	26 (52.0)
Septal	17 (34.0)
Family history	
Yes	3 (6.0)
No	10 (20.0)
unknown	37 (74.0)
Non-sustained VT	1 (2.0)
Unexplained syncope	7 (14.0)
5-year risk of SCD (%)	1.484 ± 1.031
Low-risk (risk <4%)	47 (94.0)
Intermediate risk (risk ≥4 to <6%)	3 (6.0)
Co-morbidity	
Hypertension	38 (76.0)
ßB	24
CCB	16
ACEI/ARB	19
diuretics	9
Diabetes	20 (40.0)
Hyperlipidemia	26 (52.0)
ESRD	2 (4.0)
Smoking	28 (56.0)
current smoker	13
ex-smoker	15
Cancer	4 (8.0)

Values are presented as Mean ± SD or N (%) as appropriate. ESRD, end-stage renal disease; ßB, beta blocker; CCB; calcium channel blocker; ACEI/ARB, angiotensin-converting enzyme inhibitor/angiotensin receptor blocker.

**Table 2:** Comparing three forms of HCM, and post-stress/rest data in CZT gated MPI.

Variable	All patients (n = 50)	Apical (n = 7)	Concentric (n = 26)	Septal (n = 17)	*P*_*betw*_
Age (y)	59.7 ± 12.4	59.7 ± 13.0	58.2 ± 13.7	61.9 ± 10.2	0.65
Males	25 (50.0)	6 (85.7)	11 (42.3)	8 (47.1)	0.17
Hypertension	38 (76.0)	6 (85.7)	21 (80.8)	11 (64.7)	0.52
5-year SCD risk (%)	1.484 ± 1.031	1.383 ± 1.225	1.257 ± 0.563	1.872 ± 1.395	0.14
***Variables in CZT***					
SSS [range]	4.7 ± 4.9 [0-26]	9.4 ± 9.0 [2–26]	3.5 ± 3.4 [0–12]	4.6 ± 3.4 [0–12]	0.11
0–3	28 (56.0)^§^	3 (42.9)	16 (61.5)	9 (52.9)	
4–7	10 (20.0)	1 (14.3)	6 (23.1)	3 (17.6)	
≥8	12 (24.0)	3 (42.9)	4 (15.4)	5 (29.4)	
SRS	2.8 ± 3.2	5.0 ± 4.7	2.3 ± 2.8	2.5 ± 3.0	0.16
SDS	2.0 ± 3.5	4.4 ± 5.2	1.2 ± 3.5	2.1 ± 2.4	0.40
LVEF-stress (%)	63.7 ± 14.1	55.9 ± 8.7	64.3 ± 12.4	66.1 ± 17.6	0.26
LVEF-rest (%)	62.7 ± 12.4	58.9 ± 10.0	61.9 ± 11.1	65.5 ± 15.0	0.45
*p*_*within*_	0.41	0.26	0.18	0.79	
∆ LVEF (%)	1.95 ± 14.59	−4.33 ± 11.29	4.63 ± 15.07	0.44 ± 14.77	0.31
L/H-stress	0.384 ± 0.094	0.389 ± 0.101	0.379 ± 0.094	0.391 ± 0.096	0.92
L/H-rest	0.360 ± 0.061	0.374 ± 0.083	0.356 ± 0.055	0.362 ± 0.064	0.78
*P*_*within*_	0.04*	0.65	0.18	0.13	
TPD-stress	6.5 ± 5.9	7.9 ± 10.6	5.6 ± 4.6	7.5 ± 5.4	0.52
TPD-rest	5.2 ± 5.4	6.0 ± 4.3	4.7 ± 5.2	5.8 ± 6.3	0.48
*P*_*within*_	0.07	0.93	0.22	0.15	
EDV-stress (ml)	78.7 ± 25.4	82.3 ± 21.9	77.7 ± 26.9	78.9 ± 25.6	0.78
EDV-rest (ml)	76.0 ± 25.0	75.9 ± 20.8	75.2 ± 27.5	77.4 ± 23.9	0.96
*P*_*within*_	0.14	0.17	0.31	0.62	
ESV-stress (ml)	30.9 ± 19.4	37.1 ± 13.3	30.3 ± 19.9	29.4 ± 21.1	0.32
ESV-rest (ml)	30.3 ± 16.6	32.4 ± 14.6	30.5 ± 17.9	29.2 ± 16.2	0.89
*P*_*within*_	0.78	0.13	0.86	0.96	
***Phase analysis***					
SD-stress	26.86 ± 16.90	37.47 ± 16.35	25.44 ± 17.05	24.66 ± 16.19	0.21
SD-rest	29.08 ± 12.00	34.91 ± 13.40	27.87 ± 10.00	28.51 ± 14.14	0.38
*P*_*within*_	0.11	0.66	0.12	0.25	
bandwidth-stress	85.1 ± 60.4	113.1 ± 54.6	83.4 ± 65.7	76.2 ± 53.5	0.22
bandwidth-rest	84.3 ± 38.1	100.0 ± 36.1	78.9 ± 31.7	86.1 ± 47.1	0.31
*P*_*within*_	0.48	0.44	0.46	0.26	
skewness-stress	3.47 ± 0.89	2.63 ± 0.80	3.66 ± 0.82	3.54 ± 0.86	0.03*
skewness-rest	3.13 ± 0.84	2.53 ± 0.47	3.20 ± 0.65	3.27 ± 1.11	0.08
*P*_*within*_	<0.01*	0.67	<0.01*	0.11	
kurtosis-stress	14.57 ± 8.10	7.56 ± 5.35	16.33 ± 7.81	14.75 ± 8.25	0.02*
kurtosis-rest	11.52 ± 7.43	7.27 ± 4.07	11.65 ± 5.22	13.07 ± 10.49	0.14
*P*_*within*_	<0.01*	0.87	<0.01*	0.19	

Values are presented as Mean ± SD or N (%) as appropriate. *P*_*betw*_, p value between 3 groups; *P*_*within*_, p value within stress/rest group.

^§^There were 10 patients with SSS = 3.

^#^Normal database of phase analysis from Chen *et al*.^32^ for men/women: SD (14.2 ± 5.1/11.8 ± 5.2), histogram bandwidth (38.7 ± 11.8/30.6 ± 9.6), histogram skewness (4.19 ± 0.68/4.60 ± 0.72), histogram kurtosis (19.72 ± 7.68/23.21 ± 8.16).

*p value < 0.05.

### MPI parameters

The MPI variables are summarized in Table 2. The mean summed stress score (SSS) was 4.7 ± 4.9 (range 0–26), with approximately half of the patients having an SSS ≥ 4, indicating at least mild ischemia after stress. There were no significant differences in the parameters including SSS, summed rest score (SRS), summed difference score (SDS), post-stress left ventricular ejection fraction (LVEF), resting LVEF, difference of LVEF between post-stress and rest (∆LVEF), post-stress lung-heart ratio (L/H), resting L/H, post-stress total perfusion deficit (TPD), resting TPD, post-stress end-diastolic volume (EDV), resting EDV, post-stress end-systolic volume (ESV), and resting ESV among the three groups.

For the phase analysis data, compared to the normal data established by Chen *et al*.^32^, the patients with suspected HCM had significant mechanical dyssynchrony. Post-stress skewness and kurtosis showed significant differences among the three forms, being highest in the concentric form and lowest in the apical form, suggesting less mechanical synchrony in the patients with apical HCM. In addition, although not statistically significant, most patients with the apical form showed a trend towards higher SSS, SRS, SDS, phase standard deviation (SD) and phase histogram bandwidth, as well as lower LVEF, ∆LVEF, resting phase histogram skewness and kurtosis.

Comparing post-stress data and resting data, there were no differences in LVEF, TPD, EDV, ESV, phase SD and phase histogram bandwidth. The L/H ratio was larger after stress (*P*_within_ = 0.04). In addition, phase histogram skewness and kurtosis were different between post-stress and resting status (*P*_within_ < 0.01), revealing more synchronous contraction after stress, especially in the concentric group.

### Echocardiography parameters

The echocardiographic variables are summarized in Table 3. All of the patients had a LVEF > 50% by M mode; therefore we used the normal-LVEF algorithm in the 2016 American Society of Echocardiography (ASC)/European Association of Cardiovascular Imaging (EACVI) guidelines^33^ to evaluate diastolic function. Diastolic parameters including peak velocities of transmitral flow at early filling (E) and atrial filling (A), derived E/A ratio, deceleration time (DT) at early filling, tricuspid regurgitation jet maximal velocity (peak TR velocity) and tricuspid regurgitation maximum pressure gradient (TRmaxPG) were recorded. Tissue Doppler imaging was performed in some cases, and data including peak velocities of the mitral annulus (e’) and E/e’ ratio were also acquired. Due to the retrospective nature of this study, some data required in the latest guidelines were missing, and only 38 patients could be graded. More than half of these patients (n = 22/38, 57.9%) had diastolic dysfunction, and most had mild to moderate diastolic dysfunction.

**Table 3:** Comparing resting Doppler echocardiography findings in three forms of HCM (n = 50).

Variable	All patients	apical	concentric	septal	p
LA (mm)	38.2 ± 6.5 [25–63]	40.4 ± 6.3	37.3 ± 6.5	38.8 ± 6.6	0.48
LA volume index (cm/m^2^)	27.4 ± 11.6 [10.2–57.5]	24.9 ± 10.2	24.7 ± 10.6	32.7 ± 12.4	0.08
IVS (mm)	15.7 ± 3.7 [9–27]	12.0 ± 3.4	14.9 ± 1.0	18.6 ± 4.4	<0.01*
LVPW (mm)	12.6 ± 2.2 [8–20]	12.9 ± 2.0	13.5 ± 1.4	11.3 ± 2.7	<0.01*
IVS/LVPW	1.273 ± 0.351 [0.82–2.60]	0.922 ± 0.137	1.110 ± 0.093	1.667 ± 0.309	<0.01*
LVEDD (mm)	45.9 ± 5.1 [35–56]	49.9 ± 5.3	45.2 ± 5.1	45.2 ± 4.6	0.08
LVESD (mm)	27.4 ± 4.5 [19–39]	29.6 ± 4.8	27.5 ± 4.6	26.2 ± 3.8	0.23
LVEF (%, by M mode)	70.3 ± 7.5 [51–85]	70.6 ± 6.7	69.1 ± 8.5	72.1 ± 5.9	0.43
peak TR velocity (cm/s)^$^ [n = 29]	235.1 ± 41.9 [146–316]	263.0 ± 45.9	222.7 ± 43.1	244.3 ± 36.5	0.21
TRmaxPG (mmHg)^$^ [n = 29]	22.2 ± 7.9 [8–40]	28.0 ± 10.6	19.9 ± 7.9	23.8 ± 6.5	0.19
**Mitral inflow**					
E (cm/s)	75.7 ± 19.7 [40–124]	66.0 ± 27.2	79.0 ± 18.5	74.6 ± 17.7	0.19
A (cm/s)	87.0 ± 25.3 [34–180]	75.1 ± 22.3	81.4 ± 21.7	100.5 ± 27.1	0.02*
DT (ms)	234.9 ± 69.1 [139–431]	232.4 ± 89.4	213.7 ± 48.6	268.4 ± 77.7	0.07
E/A	0.97 ± 0.50 [0.3–3.1]	1.07 ± 0.94	1.05 ± 0.42	0.80 ± 0.34	0.03*
**Septal mitral annulus**					
e’ (cm/s)^Φ^ [n = 33]	5.66 ± 1.94 [2.4–9.9]	5.84 ± 2.05	5.72 ± 1.78	5.46 ± 2.34	0.93
a’ (cm/s)^#^ [n = 21]	8.43 ± 2.11 [3.7–13.0]	8.57 ± 1.23	8.73 ± 2.10	6.80 ± 2.72	0.37
E/e’^Φ^ [n = 33]	15.174 ± 6.315 [7.70–37.50]	12.440 ± 3.414	15.171 ± 5.507	16.548 ± 8.550	0.53
e’/a’^#^ [n = 21]	0.651 ± 0.211 [0.34–1.21]	0.713 ± 0.432	0.660 ± 0.180	0.547 ± 0.100	0.54
***diastolic dysfunction grade***					
**(1) by 2016 guideline (n = 38)**					0.21
Normal	16 (42.1)	4	9	3	
Mild/impaired relaxation	9 (23.7)	0	5	4	
Moderate/pseudonormalization	10 (26.3)	0	5	5	
Severe/restrictive filling	3 (7.9)	1	2	0	
**(2) by mitral flow (n = 50)**					0.03*
Normal	9 (18.0)	1 (14.3)	7 (26.9)	1 (5.9)	
Mild/impaired relaxation	19 (38.0)	5 (71.4)	6 (23.1)	8 (47.1)	
Moderate/pseudonormalization	19 (38.0)	0	11 (42.3)	8 (47.1)	
Severe/restrictive filling	3 (6.0)	1 (14.3)	2 (7.7)	0	

Values are presented as Mean ± SD [range] or N (%) as appropriate. IVS, interventricular septum; PW, posterior wall; EDD, end-diastolic dimension; ESD, end-systolic dimension; e’, early peak velocity of the septal mitral annulus during atrial contraction; a’, late peak velocity of the septal mitral annulus during atrial contraction.

^$^Three cases in apical form, fifteen cases in concentric form, and eleven cases in septal form.

^Φ^Five cases in apical form, eighteen cases in concentric form, and ten cases in septal form.

^#^Three cases in apical form, fifteen cases in concentric form, and three cases in septal form.

*p value < 0.05.

If we graded the cases according to the mitral inflow measurements detailed in the Methods section, 82% (n = 41/50) of the included patients had diastolic dysfunction. Most of the patients presented with mild and moderate dysfunction. The grading of diastolic dysfunction among the three forms was statistically significantly different: the apical form showed mostly impaired relaxation, the septal form had either impaired relaxation or pseudonormalization, and the concentric form had various levels of dysfunction.

The means of peak TR velocity and left atrial volume index (LAVI) were within normal range. However, although incomplete, the means of tissue Doppler imaging variables suggested diastolic dysfunction: most septal e’ (24/33 = 72.7%) values were less than 7, and almost half of the septal E/e’ (15/33 = 45.5%) values were larger than 15. Except for A velocity and E/A ratio, no differences were found among the three groups.

### Correlations

**Figure 1:**
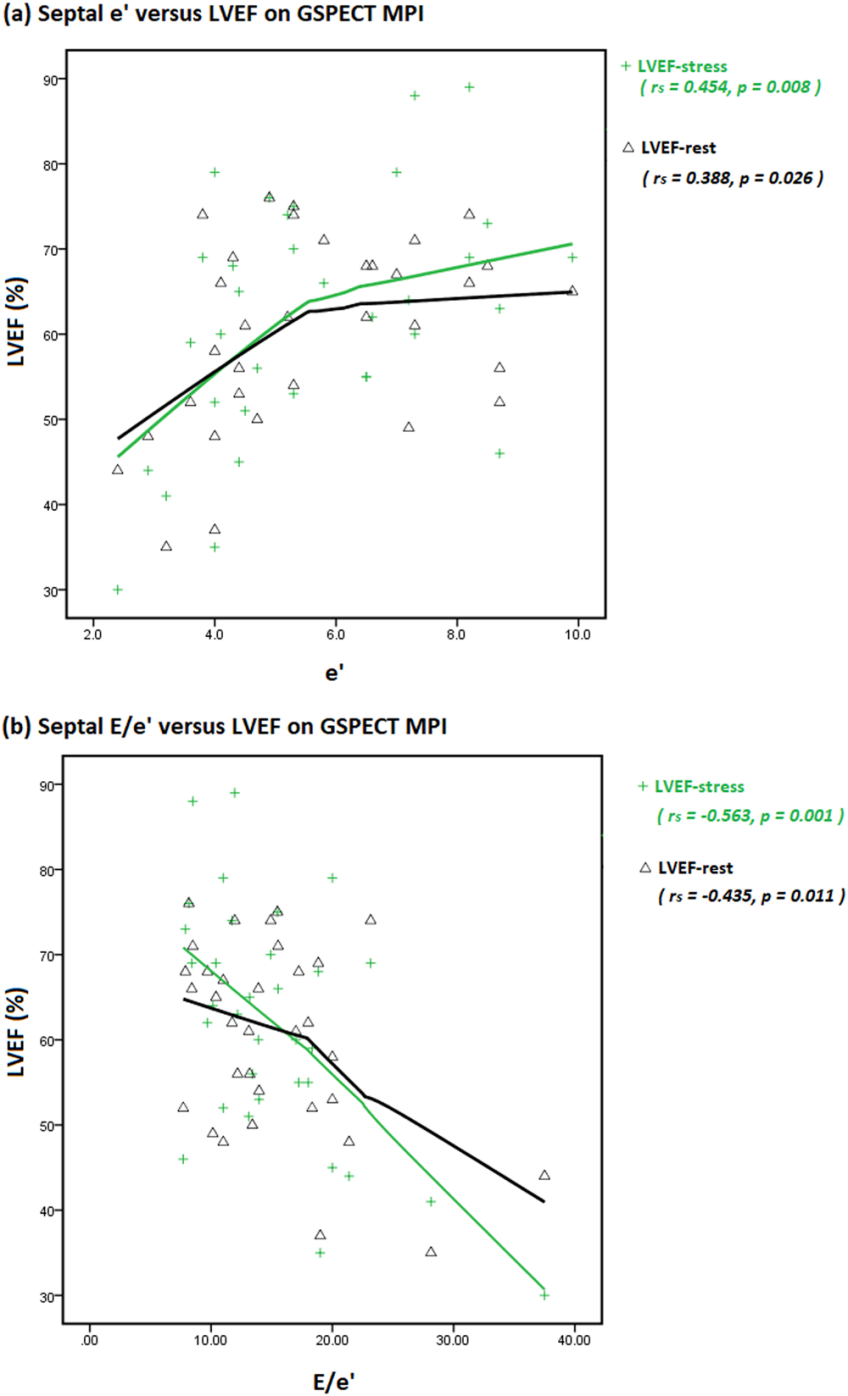
Positive Spearman’s rank correlations were seen between (**a**) e’ and LVEF on GSPECT MPI, and negative Spearman’s rank correlations between (**b**) E/e’ and LVEF on GSPECT MPI; both in post-stress and in resting status.

There were no significant correlations among E/A ratio, DT, LAVI and GSPECT MPI variables. Table 4 shows the significant correlations between GSPECT MPI parameters and diastolic variables on Doppler echocardiography. Septal e’ was positively correlated with post-stress LVEF, resting LVEF (Fig. 1a) and resting L/H. Septal E/e’ was negatively correlated with post-stress LVEF, resting LVEF (Fig. 1b), ∆LVEF and resting L/H, and positively correlated with post-stress ESV. Peak TR velocity was positively correlated with resting L/H, and negatively correlated with post-stress histogram skewness.

**Table 4:** Correlations between GSPECT MPI parameters and diastolic variables on Doppler echocardiography.

	Septal e’ (n = 33)		Septal E/e’ (n = 33)		peak TR velocity (n = 29)	
	Correlation coefficient	P value	Correlation coefficient	P value	Correlation coefficient	P value
LVEF-stress	0.454	0.008*	−0.563	0.001*	−0.014	0.941
LVEF-rest	0.388	0.026*	−0.435	0.011*	0.085	0.661
∆ LVEF	0.218	0.222	−0.366	0.036*	−0.174	0.368
ESV-stress	−0.341	0.052	0.404	0.020*	0.061	0.753
L/H-rest	0.474	0.005*	−0.350	0.046*	0.442	0.016*
Skewness-stress	0.216	0.228	−0.284	0.110	−0.381	0.042*

*p value < 0.05.

Table 5 shows the significant relationships between interventricular septal (IVS) thickness, LV posterior wall (LVPW) thickness, IVS/LVPW ratio and GSPECT MPI variables. IVS thickness was negatively correlated with LVEF and resting L/H, and positively correlated with post-stress EDV, and post-stress and resting ESV. In addition, LVPW thickness was negatively correlated with LVEF, post-stress histogram skewness and kurtosis, and positively correlated with post-stress EDV, post-stress and resting ESV, phase SD and histogram bandwidth. However, no significant correlations were noted between IVS/LVPW ratios and the GSPECT MPI variables.

**Table 5:** Correlations between MPI parameters and myocardial wall thickness and derived ratio (n = 50).

	IVS		LVPW		IVS/LVPW	
	Correlation coefficient	P value	Correlation coefficient	P value	Correlation coefficient	P value
LVEF-stress	−0.345	0.014*	−0.434	0.002*	−0.001	0.993
LVEF-rest	−0.368	0.009*	−0.462	0.001*	−0.002	0.988
∆ LVEF	−0.084	0.560	−0.053	0.714	−0.030	0.836
EDV-stress	0.432	0.002*	0.287	0.043*	0.020	0.890
EDV-rest	0.251	0.079	0.129	0.371	0.146	0.311
ESV-stress	0.289	0.042*	0.353	0.012*	−0.135	0.351
ESV-rest	0.361	0.010*	0.305	0.031*	−0.002	0.990
TPD-stress	0.155	0.282	0.067	0.642	0.110	0.448
TPD-rest	0.106	0.465	0.225	0.116	−0.049	0.735
L/H-stress	−0.197	0.171	−0.091	0.529	−0.105	0.468
L/H-rest	−0.331	0.019*	−0.115	0.425	−0.213	0.137
SD-stress	0.235	0.100	0.339	0.016*	−0.173	0.231
Bandwidth-stress	0.244	0.088	0.366	0.009*	−0.197	0.170
Skewness-stress	−0.090	0.536	−0.350	0.013*	0.188	0.190
Kurtosis-stress	−0.190	0.186	−0.303	0.032*	0.191	0.184
SD-rest	0.178	0.217	0.307	0.030*	−0.180	0.210
Bandwidth-rest	0.230	0.108	0.326	0.021*	−0.157	0.276
Skewness-rest	−0.105	0.470	−0.114	0.430	0.141	0.330
Kurtosis-rest	−0.048	0.743	−0.037	0.800	0.123	0.395

*p value < 0.05.

## Discussion

Myocardial ischemia is commonly seen in patients with HCM, even in those with patent coronary arteries, especially after stress. Myocardial ischemia can predict unfavorable disease, and can be attributable to either microvascular dysfunction or increased LV mass^15,34^. Decreased myocardial blood flow and LVEF reserve seen on positron emission tomography (PET) studies after dipyridamole stress also implies that subendocardial ischemia can cause transient LV dysfunction^35^.

Only one study^36^ has discussed HCM with phase analysis, and it compared LV dyssynchrony in patients with HCM before and after alcohol septal ablation. However, they did not mention the HCM classification and only used resting GSPECT MPI. Moreover, the imaging was acquired with conventional gamma cameras with Tc-99 m sestamibi.

To the best of our knowledge, this is the first GSPECT MPI study to assess patients with HCM using a CZT camera with a stress test. Our results reconfirmed that HCM was associated with myocardial ischemia and LV dyssynchrony. Apical HCM was associated with lower post-stress skewness and kurtosis, and the concentric form had the highest post-stress skewness and kurtosis, suggesting more prominent post-stress LV dyssynchrony in apical HCM and more synchronous contraction in the concentric form. On the other hand, although there was a trend that apical HCM was more dyssynchronous in all phase parameters, phase SD and phase histogram bandwidth were not significantly different among the three groups. This could imply that the differences were relatively small, and due to less statistically different phase data between the concentric and septal forms.

When we compared the “post-stress” and “resting” data, a larger L/H ratio was seen after stress. This may imply exercise-induced elevations in pulmonary capillary wedge pressure, LV systolic and perhaps diastolic dysfunction^37^. In addition, higher skewness and kurtosis were noted after stress, especially in the concentric form. Chen *et al*.^38^ discussed similar observations in 2012. They found that LV dyssynchrony was more prominent after stress in ischemic myocardium, but that significantly smaller LV dyssynchrony was noted after stress in normal and infarcted myocardium. In other words, unlike ischemic myocardium, stress could cause more synchronous contraction in normal (SSS < 5, SRS < 5) and infarcted myocardium^38^. The patients in our study had SSS 4.7 ± 4.9 and SRS 2.8 ± 3.2 (SSS 3.5 ± 3.4 and SRS 2.3 ± 2.8 in the concentric form), which were closer to the normal myocardium and could indicate milder disease severity.

In the current study, only patients with a LVEF > 50% were enrolled. According to Smiseth *et al*.^39^, there are three hallmarks in the evaluation of diastolic dysfunction: (1) impaired relaxation, (2) loss of restoring forces, and (3) increased diastolic stiffness. The prior two features could be reflected by a decreased e’^39,40^, and increased stiffness/poor compliance could be manifested as a high E/A ratio and decreased DT (i.e. grade III diastolic dysfunction)^39^. Based on this mitral flow pattern classification, 82% (n = 41) of the patients had diastolic dysfunction, and mitral flow and E/A ratios were significantly different between the three forms of HCM. E/e’, peak TR velocity and LAVI have been associated with left atrial (LA) pressure and have been used to estimate LV filling pressure^39,40^. We analyzed 38 patients based on the “normal LVEF algorithm” of the 2016 ASC/EACVI guidelines^33^, and more than half (22/38 = 57.9%) had diastolic dysfunction. E/e’ was positively correlated with post-stress ESV, suggesting that increased resting LV filling pressure could predict transient stress-induced LV dilatation. In addition, peak TR velocity, a commonly used echocardiographic estimation of systolic pulmonary artery pressure, was negatively correlated with post-stress phase histogram skewness, suggesting a relationship with LV dyssynchrony.

The severity of LV dyssynchrony has been linked to the magnitude of LVH and possible diastolic dysfunction either with or without heart failure, and HCM has been shown to be more prominent in LV dyssynchrony than in HCVD^41,42^. Our results confirmed that the thickness of the LV myocardial wall (either septal or free wall) was associated with LVEF impairment, LV volume dilatation and LV dyssynchrony.

The L/H ratio may reflect pulmonary capillary wedge pressure, and a higher L/H ratio has been associated with LV dysfunction^43,44^. For thallium-201, the reported upper limit ranges from 0.37 to 0.55^45,46^. The L/H ratios of both post-stress and resting phases in our cohort were within normal range. Our results showed controversial correlations between resting L/H ratio and diastolic function: with a positive correlation with peak TR velocity; but with negative correlation with E/e’, and positive correlation with e’. This may be due to the limited number of cases. However, we cannot explain the negative correlation between IVS thickness and resting L/H ratio, although the correlation was weak and was not seen for LVPW and post-stress L/H ratio.

### Limitations

The major limitations to this study are related to its retrospective design. The patients’ clinical severity differed, but only those with patent coronary arteries and those who underwent both Doppler echocardiography and GSPECT MPI were enrolled. We did not exclude the patients with underlying hypertension, and those with incomplete family history information, and thus patients with HCVD may have been included, and the 5-year risk of SCD may have been underestimated.

Although variability has been described between operators, different ultrasound machines and software when measuring tissue velocities^47^, echocardiographic velocity measurements can be more consistent than previously described, and it was clinically feasible to apply the 2016 ASC/EACVI recommendations in this cross-sectional study. However, not all of the patients had complete information regarding the latest guidelines for evaluating diastolic dysfunction, and therefore only 38 (76%) patients could be graded accordingly. Finally, this was a single-center study with a limited number of cases, and the patients with significant arrhythmia, conduction abnormalities, severe valvular disease, or other cardiomyopathies, thus extrapolation of the results to the general population needs further investigations.

## Methods

### Study population

This study was approved by the Institutional Review Board of Far Eastern Memorial Hospital (106057-E). The need for written informed consent was waived due to the retrospective nature of the study. All procedures and methods were performed in accordance with the updated guidelines and regulations.

All patients who underwent cardiac catheterization between January 2013 and October 2016 were reviewed, and those with significant (≥50%) coronary stenosis were excluded. The transthoracic Doppler echocardiography and CZT gated SPECT MPI data within 6 months were analyzed. Patients with either a spade-like configuration on invasive left ventriculogram or findings indicative of HCM on echocardiography (i.e., an LV wall thickness ≥14 mm) were enrolled.

The patients with a history of significant coronary stenosis (≥50%), myocardial infarction, coronary artery bypass grafting or percutaneous coronary intervention, severe valvular disease, significant arrhythmia (including atrial fibrillation, atrial flutter, frequent atrial and ventricular arrhythmia), conduction disorders (including sick sinus rhythm, second and third degree atrioventricular block, pacing rhythm, and left bundle branch block), documented congenital heart disease, dilated or restrictive cardiomyopathy, Kawasaki disease, or pulmonary embolism were excluded. All of the included patients had sinus rhythm during Doppler echocardiography and GSPECT MPI. Although some patients were comorbid with hypertension, all patients were well-controlled (SBP ≦ 150 mmHg) in the MPI studies, and none was diagnosed with resistant hypertension.

**Figure 2:**
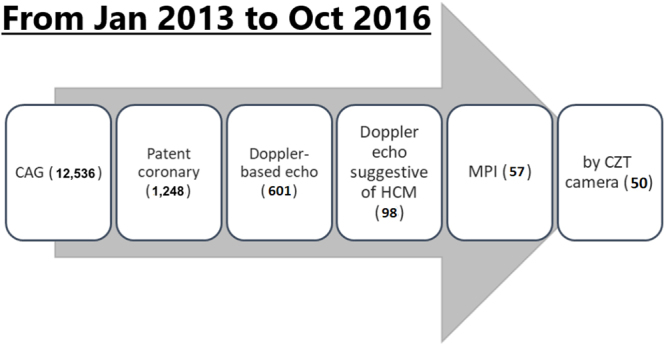
The inclusion algorithm of the study population. A total of 50 patients with both CZT-based MPI and Doppler echocardiography were included. CAG, coronary angiography; echo, echocardiography.

Demographic data were collected from medical records, including age, gender, height, weight, cardiovascular risk factors, major systemic diseases and medications, documented arrhythmia, and family history of SCD. To calculate the 5-year risk of SCD based on the 2014 European Society of Cardiology guidelines^11^, a history of unsustained ventricular tachycardia (VT) and unexplained syncope was also recorded. Figure 2 shows the inclusion algorithm. The enrolled patients were then divided into three groups: those with apical, concentric and septal forms. The concentric and septal forms were discerned according to the ratio of interventricular septum to free wall, and defined as the septal form if the ratio was ≥1.3.

### Echocardiography

Resting Doppler echocardiographic studies were performed using either a GE VIVID 7 (GE Healthcare, Chicago, Illinois, USA), GE VIVID 9 (GE Healthcare, Chicago, Illinois, USA), or Philips iE 33 (Philips Healthcare, Cleveland, Ohio, USA) echocardiography system. The frame rate was set as default, with the GE VIVID at around 100/s and the Philips iE 33 in the range of 70–130/s. Echocardiography was performed by experienced cardiologists, and data including M mode and two-dimensional Doppler imaging were collected. LA and LV dimensions and related indexes were measured. E, A, E/A ratio, DT, peak TR velocity, TRmaxPG, also tissue Doppler imaging variables (e’, E/e’) in some cases, were included for diastolic function analysis. To reduce the effect of heart rate variability, each tissue Doppler image was acquired at least twice, and peak velocity measurements were averaged across acquisitions and multiple beats.

According to the latest recommendations by the ASC and EACVI^33^ published in 2016, four variables should be considered when considering diastolic dysfunction: (a) septal e’ < 7 cm/s or lateral e’ < 10 cm/s, (b) average E/e’ > 14 (either lateral E/e’ > 13 or septal E/e’ > 15), (c) LAVI > 34 ml/m^2^, and (d) peak TR velocity > 280 cm/s. If more than half of the available parameters meet the cutoff values, diastolic dysfunction is present; if half of the available parameters meet the cutoff values, it is inconclusive; and if less than half of the available parameters meet the cutoff values, diastolic function is normal. Diastolic dysfunction was then graded using the algorithm published in the 2016 ASC/EACVI guidelines into (1) normal, (2) mild dysfunction/impaired relaxation, (3) moderate dysfunction/pseudonormalization, and (4) severe dysfunction/restrictive filling.

Due to the retrospective nature of the study, some diagnostic variables needed in the latest guidelines were not available. Thus we also graded the cases on the basis of mitral inflow as follows: (1) normal (E/A > 0.8 to < 2, DT > 150 ms, ±septal E/e’ < 11); (2) mild dysfunction/impaired relaxation (E/A ≤ 0.8, DT > 150 ms); (3) moderate dysfunction/pseudonormalization (E/A > 0.8 to < 2, DT > 150 ms, ±septal E/e’ ≥ 11); and (4) severe dysfunction/restrictive filling (E/A ≥ 2, DT < 150 ms)^48–50^.

### Dipyridamole electrocardiogram-gated MPI protocol

Each patient underwent dipyridamole-stress and rest GSPECT MPI using thallium-201 (Tl-201) with a CZT camera. Dipyridamole was intravenously infused at 0.56 mg/kg over a 4-minute period. A dose of 2 mCi (74 MBq) Tl-201 was then injected at the 7^th^ minute. The injected dose was 2.5 mCi (92.5 MBq) if the patient weighed more than 90 kg, and 3.0 mCi (111 MBq) if the patient weighed more than 100 kg. At the 10^th^ minute, an intravenous injection of 75–125 mg of aminophylline was administered to prevent/treat dipyridamole-induced adverse effects. Imaging acquisition began within 5 minutes after the aminophylline injection and was repeated 4 hours later. The CZT gamma camera (Discovery NM 530c, GE Healthcare, Chicago, Illinois, USA) was equipped with 19 pinhole collimators and 19 solid-state CZT detectors along a 180 degree arc. Each detector was 8 × 8 cm in size, and consisted of 32 × 32 (2.46 × 2.46 mm in size) pixilated components. The energy window was set as default for Tl-201: asymmetrically (−14% to +23%) at 70 keV, and symmetrically (−9% to +9%) at 167 keV. Electrocardiogram-gating was also implemented using a built-in system^48,51,52^.

### MPI parameters

The stress/rest images were read by two experienced nuclear medicine physicians who were unaware of the clinical, angiographic, and echocardiographic data. Quantitative perfusion SPECT/quantitative gated SPECT (QPS/QGS) software (Cedars-Sinai Medical Center, Los Angeles, California, USA) was used for GSPECT MPI analysis.

GSPECT MPI can display pathophysiologic changes and evaluate both LV myocardial perfusion and function in one-step^28^. Regional perfusion was analyzed using a 17-segment model and graded from 0–4 (0 = normal perfusion; 1 = mild defect; 2 = moderate defect; 3 = severe defect, 4 = no perfusion), and SSSs, SRSs and SDSs were determined according to the standards detailed above. Myocardial functional data were also derived, including LVEF and phase information.

Initially proposed in the 1990s, phase analysis is a well-established method to assess dyssynchrony of the LV myocardial wall^32,53,54^. By equally dividing every R-R interval into eight frames, counts of each frame can be measured; harmonic Fourier transformation is then used to estimate the onset of mechanical contraction of each region, which is then displayed as phase angle^32,53^. Phase analysis provides information regarding homogeneity of the onset of the mechanical contraction of each region in the entire left ventricle; in other words, the synchrony of LV^32^.

**Figure 3:**
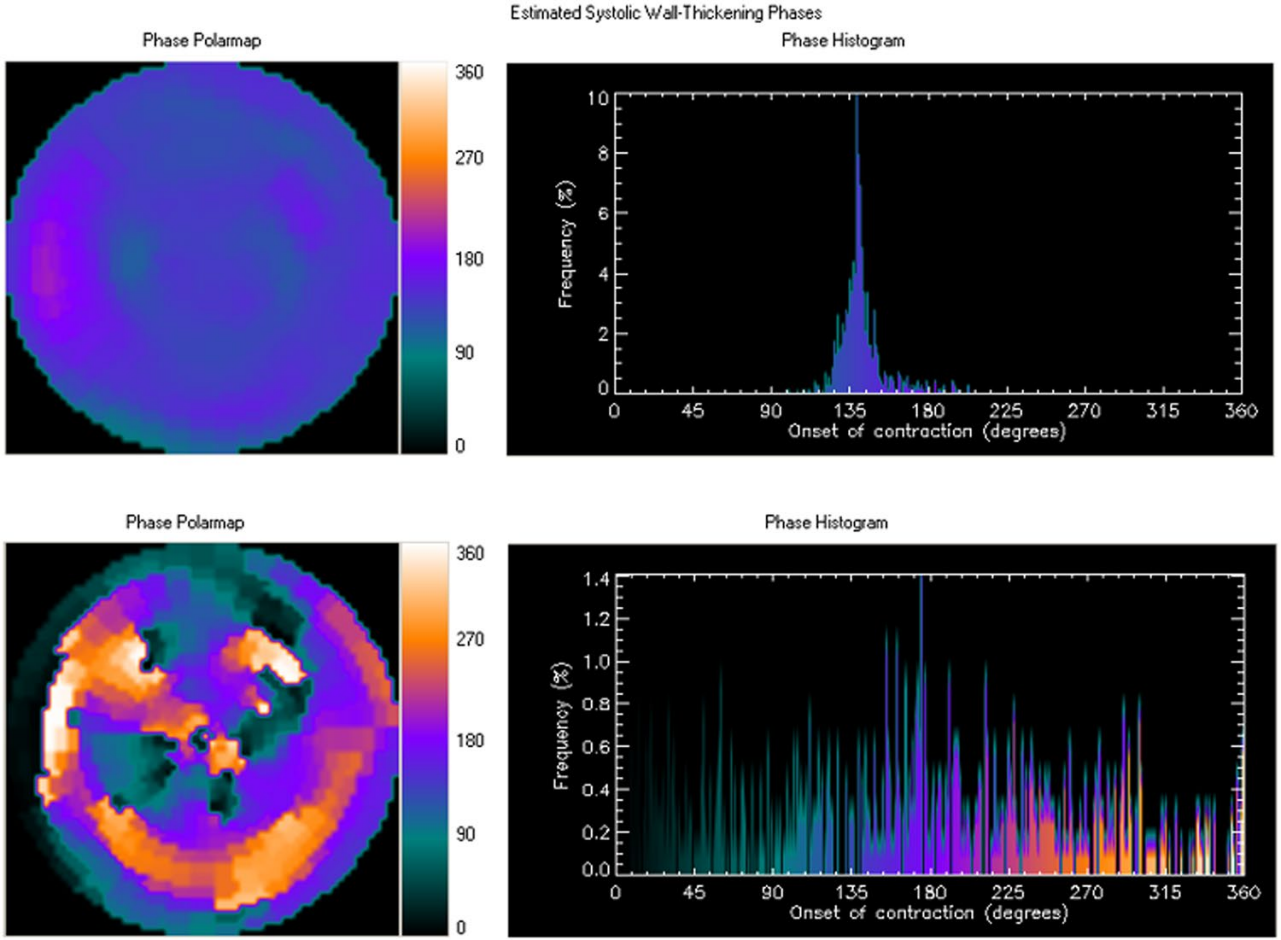
The representative examples of phase polar maps and phase histograms in patients without (upper panel) and with LV dyssynchrony (lower panel).

Phase histograms and parameters were automatically derived using QGS software. Figure 3 shows representative examples of histograms of patients without (upper panel) and with LV dyssynchrony (lower panel). The four phase parameters included phase SD, phase histogram bandwidth, phase histogram skewness, and phase histogram kurtosis. Phase SD represents the SD of phase distribution; phase histogram bandwidth includes 95% of the left ventricle myocardium; phase histogram skewness demonstrates the symmetry of the histogram; and phase histogram kurtosis reflects how the histogram is peaked^29,55^. In the less synchronous left ventricle, a larger phase SD and histogram bandwidth can be observed, as well as smaller histogram skewness and histogram kurtosis^29,55,56^. Researchers have also established normal data as follows^32^: phase SD, 14.2 ± 5.1 degrees for men and 11.8 ± 5.2 degrees for women; phase histogram bandwidth, 38.7 ± 11.8 degrees for men and 30.6 ± 9.6 degrees for women; phase histogram skewness, 4.19 ± 0.68 for men and 4.60 ± 0.72 for women; and phase histogram kurtosis, 19.72 ± 7.68 for men and 23.21 ± 8.16 for women. Several phase analysis studies have discussed conduction and mechanical diseases (e.g., left bundle branch block and coronary artery disease), cardiac resynchronization therapy (CRT), ventricular tachyarrhythmia genesis, chronic kidney disease, and heart failure^28,29,54–60^.

### Statistical analysis

Numerical variables were expressed as mean ± SD, and categorical variables were expressed as percentages. Differences between numerical variables were compared using the paired-T test and one-way ANOVA for parametric data, and Wilcoxon signed-rank test and Kruskal-Wallis test for nonparametric data, as appropriate. Differences between categorical variables were compared using Fisher’s exact test. To determine interrelationships between two variables from echocardiography and GSPECT MPI, Pearson’s correlation analysis was used if the data were parametric, and Spearman’s rank correlation if the data were nonparametric.

Comparisons were made between post-stress and resting data and among the three different forms of LVH. Data were analyzed using SPSS software version 22.0 (IBM Corp., Armonk, New York, USA). All *p-*values were 2-sided, and a *p*-value < 0.05 was considered to be statistically significant.

### Data availability

The data are available from the corresponding author on reasonable request.

### IRB

FEMH 106057-E (Far Eastern Memorial Hospital).

## Conclusion

By combining GSPECT MPI and Doppler data, we showed that LVH in patients with suspected HCM was associated with myocardial ischemia, LV mechanical dyssynchrony and diastolic dysfunction. We also found that dyssynchrony was slightly different among the three forms of HCM, and the correlations could help demonstrate trivial functional changes.
